# Inactivation and Removal of Chikungunya Virus and Mayaro Virus from Plasma-derived Medicinal Products

**DOI:** 10.3390/v11030234

**Published:** 2019-03-07

**Authors:** Constanze Yue, Sebastian Teitz, Tomoyuki Miyabashi, Klaus Boller, Lia Laura Lewis-Ximenez, Sally A. Baylis, Johannes Blümel

**Affiliations:** 1 Department of Virology, Paul-Ehrlich-Institut, 63225 Langen, Germany; Klaus.Boller@pei.de (K.B.); Sally.Baylis@pei.de (S.A.B.); Johannes.Bluemel@pei.de (J.B.); 2 Asahi Kasei, 51105 Cologne, Germany; s.teitz@akbio.eu (S.T.); miyabayashi.tb@om.asahi-kasei.co.jp (T.M.); 3 Instituto Oswaldo Cruz, Fundaçăo Oswaldo Cruz, Rio de Janeiro, RJ 21040-900, Brazil; lialewis.fiocruz@gmail.com

**Keywords:** alphaviruses, inactivation, heat treatment, S/D treatment, plasma-derived medicinal products, chikungunya virus, Mayaro virus, virus retentive filtration, nanofiltration

## Abstract

Background: Chikungunya virus (CHIKV) and Mayaro virus (MAYV) are closely related members of the Semliki Forest complex within the genus *alphavirus* and are transmitted by arthropods, causing acute febrile illness in humans. CHIKV has spread to almost all continents, whereas autochthonous MAYV infections have been reported in South America and in the Caribbean. Nevertheless, there was concern about potential spread of MAYV to other regions similar to CHIKV in the past. The risk for transmission of emerging viruses by blood transfusion and the safety of plasma-derived medicinal products (PDMPs) are constant concerns. The manufacturing processes of PDMPs include procedures to inactivate/remove viruses. Methods: In this study, we investigated the reduction of MAYV and CHIKV by heat inactivation in various matrices, solvent/detergent treatment and nanofiltration. Results: Unexpectedly, MAYV was significantly more resistant to heat and solvent/detergent treatment compared to CHIKV. However, being similar in size, both MAYV and CHIKV were removed below the detection limit by 35 nm virus filters. Conclusions: The inactivation profiles of different *alphavirus* members vary considerably, even within the Semliki Forest Complex. However, robust dedicated viral inactivation/removal procedures commonly used in the plasma product industry are effective in inactivating or removing MAYV and CHIKV.

## 1. Introduction

Chikungunya virus (CHIKV) and Mayaro virus (MAYV) belong to the Semliki Forest complex within the genus *alphavirus* and the family *Togaviridae*. Alphaviruses are positive-sense, single-stranded RNA viruses with a 11–12 kilobase genome. The enveloped particles are spherical with a diameter of 60–70 nm [1,2,3]. Like the other members of the Semliki Forest complex, o’nyong-nyong virus, Ross River virus, and Semliki Forest virus (SFV), CHIKV and MAYV are zoonotic viruses transmitted to humans by arthropods. The occurrence of alphavirus infections in humans depends on the arthropod vector and the vertebrate hosts of the respective virus. Whereas MAYV is transmitted by *Haemagogus* mosquitoes and infections in humans have been detected so far in South and Central America and the Caribbean [4], CHIKV is transmitted by *Aedes aegypti* and *Aedes albopictus*, spreading the disease to countries in Africa, Asia, Europe, and the Indian and Pacific Oceans [5]. The symptoms of a MAYV infection are generally mild and self-limiting and include fever, myalgia, headache, and rash, occasionally vomiting, abdominal pain and arthralgia—the latter can persist up to 2 months [6]. Considering its potential transmission by urban mosquitoes such as *Aedes aegypti* in experimental studies, MAYV could become a more widespread pathogen as happened in the past for CHIKV [7,8]. No vaccines or therapeutics exist for either virus and, both viruses are classified as biosafety level 3 agents.

CHIKV causes similar symptoms to MAYV, but the infection manifests in a more severe and longer-lasting course of disease. The resemblance of the clinical signs of alphavirus infections and the similarity to those of tropical diseases caused by viruses such as dengue and Zika fever hamper the diagnosis resulting in misdiagnosis and underestimation of case numbers [9]. The close phylogenetic relationship amongst members of the Semliki Forest complex complicates specific antibody detection. As a consequence, few alphavirus-specific diagnostic kits are commercially available, some of them showing inevitable cross-reactivity [10], in particular between MAYV, CHIKV and o’nyong-nyong virus [11,12]. Common serological assays such as plaque reduction neutralization tests (PRNT_50_) or reduction of cytopathic effects in a 96-well plate format (tissue culture infectious dose, TCID_50_), which both detect antibodies specifically neutralizing infectious virus particles, require pre-treatment of the plasma/serum samples at 56 °C for 30 min to inactivate complement and adventitious viruses which could interfere with the serological test system.

Besides the natural transmission of alphaviruses by mosquito species, the transmission of emerging viruses by blood transfusion or plasma-derived medicinal products (PDMPs) is a matter of concern in the public health community. Thus, infection with arboviruses might have consequences for the blood transfusion services, as seen with Zika virus (ZIKV) in Brazil [13], West Nile virus (WNV) in the US since 2002 [14,15], CHIKV on La Réunion Island [16] and in Italy in 2007 and 2017 [17,18], and WNV and Usutu virus in Europe [19,20]. Established methods for the inactivation or removal of viral pathogens in plasma pools include pasteurization—10 h at 60 °C—, solvent/detergent (S/D) treatment and virus retentive filtration (nanofiltration). Only a few studies investigating the inactivation of alphaviruses including CHIKV, Sindbis virus (SINV) and SFV in PDMPs by heat or S/D treatment have been published [21,22,23,24,25,26,27,28,29]. In this study, we investigated the inactivation/removal of MAYV and CHIKV by heat inactivation in different matrices such as medium and albumin solutions. In addition we evaluated S/D treatment using different combinations of S/D reagents. Finally, we compared the virion size by electron microscopy and investigated the virus removal by filtration through filters of differing nominal pore size.

## 2. Materials and Methods

### 2.1. Viruses and Cells

African green monkey kidney cells (Vero E6, ATTC CRL-1586) were cultivated in Dulbecco’s Modified Eagle’s Medium (DMEM) supplemented with 5% fetal bovine serum (FBS) and antibiotics. CHIKV strain BR RK430 was isolated on Vero E6 cells from the serum of a Brazilian patient, whose blood was drawn in May 2016, followed at the Viral Hepatitis Ambulatory/FIOCRUZ/Rio de Janeiro following approval by the local institutional review board approval of study amendment, approval date May 10, 2016 (Fiocruz IRB ID: 0142/01). The patient tested positive for CHIKV RNA in 2016. MAYV strain TC652 was obtained from the Public Health England Culture Collections (ECACC No. 0906281v). CHIKV and MAYV were propagated on Vero E6 cells and harvested from the cell culture supernatant at 2–3 days post infection when a cytopathic effect was observed. The harvest fluid was cleared from cellular material by low speed centrifugation and stored at −80 °C. All experiments with infectious CHIKV or MAYV were performed in the BSL-3 facilities at the Paul-Ehrlich-Institute.

### 2.2. Virus Titration

Infectious CHIKV or MAYV particles were quantified on Vero E6 cells by endpoint titration. Sub-confluent cells were inoculated with 50 µL of a 1:5 dilution series of CHIKV or MAYV solution (in untreated controls or in samples treated for virus inactivation/removal) in a 96-well plate. After incubation for 1 h at 37 °C/5% CO_2_, 150 µL DMEM containing 2% FBS was added per well. The cytopathic effect (CPE) in individual wells was assessed by microscopy after 3–5 days. The 50% tissue culture infectious dose (TCID_50_) was calculated by maximum likely algorithm [30,31] using the software CombiStats 5.0 (European Directorate for the Quality of Medicines & Healthcare, Strasbourg). Large volume plating was performed to extend the sensitivity of the assay [32,33]. In brief, during the virus inactivation/removal experiments, two 96-well plates were inoculated with the pre-diluted samples taken at each time point. At the end of each virus inactivation/removal experiment, two additional 96-well plates were inoculated with the processed sample. Due to the large volume, which is screened for infectious virus, the sensitivity of the assay is considerably increased compared to a standard titration.

To rule out that the observed CPE is caused by cytotoxic effects of the test matrix rather than by viral replication, cytotoxicity controls were performed by inoculation of cells with several dilutions of the test matrix [34]. For the determination of interference of the test matrix with the virus detection, viral infectivity in cell culture medium was compared to the viral infectivity in serial dilutions of the test matrix.

### 2.3. CHIKV RNA Detection

Before RNA extraction, the samples were treated with nuclease to remove non-encapsidated genomic virus RNA as previously described [34]. After nuclease treatment, extraction of RNA was performed from 200 µL of the sample matrix using the virus spin kit (QIAamp MinElute, Qiagen GmbH, Hilden, Germany) according to the manufacturer’s protocol. Nucleic acid elution was performed using 70 µL of elution buffer, and 5 µL of the eluate was used for the reverse transcription-PCR (RT-PCR). For the detection of CHIKV RNA, amplification reactions were performed using the RealStar Chikungunya RT-PCR Kit 2.0 (altona Diagnostics GmbH, Hamburg, Germany) according to the manufacturer’s protocol. Samples were assayed at least in duplicate. Results were expressed as IU/mL using the WHO 1st CHIKV International Standard (code number 11785/16) [35]. Detection of viral RNA was performed using a real-time PCR system (CFX96, Bio-Rad Laboratories, Inc., California, USA) in accordance with the manufacturer’s instructions.

### 2.4. Heat Inactivation in Cell Culture Medium and Human Serum Albumin Solution

Medium (DMEM without serum) was preheated to 56 °C in a water bath. Commercial 5% and 25% human serum albumin (HSA) solution was heated to 58 °C in a water bath. The temperature was measured throughout the experiment by monitoring a non-spiked medium sample incubated in parallel using a calibrated thermometer. Frozen virus stocks were thawed and pre-filtered using a 0.45 µm filter, to remove viral aggregates. The 40 mL of preheated DMEM or albumin solution was spiked at a ratio of 1:10 with CHIKV or MAYV and incubated at 56 °C or 58 °C, respectively. Samples were withdrawn after 5, 15, 30, 60, 90 and 120 min of heat treatment. Samples were immediately diluted 1:5 in cold cell culture medium and titrated on Vero E6 cells. The studies were performed in triplicate unless otherwise specified. Virus titer was determined by TCID_50_ assay. Hold controls were performed by spiking of the test matrix at room temperature and titration at the beginning and at the end of the experiment.

### 2.5. Solvent/Detergent Treatment

Virus inactivation kinetics were determined with three mixtures of S/D reagents. Final concentrations of either 0.3% tri-n-butyl phosphate (TNBP)/1% Triton X-100, 0.3% TNBP/1% Tween 80 or 0.3% TNBP/0.2% sodium deoxycholate were used. Commercial 5% HSA was mixed with S/D reagents and adjusted to 27 °C in a water bath for treatment with TNBP/Triton X-100 or TNBP/Tween 80 or 29 °C for treatment with TNBP/sodium deoxycholate. CHIKV or MAYV was added at a 1:10 ratio and mixed thoroughly. Samples were taken after 5, 15, 30, 60, 90, 120 and 150 min. The reactions were stopped by 100-fold dilution into cell culture medium. Virus titers were determined by TCID_50_ assay. Bench controls were performed by spiking of the HSA without the addition of S/D reagents and titration at the beginning and at the end of each experiment.

### 2.6. Nanofiltration

A 0.5% HSA solution was spiked 1:10 with CHIKV or MAYV and processed through 0.1 µm syringe filters (Sartorius, Göttingen, Germany). The pre-filtered solution was then processed through a 75 nm, 35 nm, or 20 nm virus filter (Asahi Kasei Bioprocess, Tokyo, Japan). In another experiment, 100 mL of virus-spiked 0.5% albumin was processed consecutively through a series of Planova filters with different average pore diameters, starting with 75 nm (Planova 75N) and followed by 40 nm, 35 nm (Planova 35N), and 20 nm (Planova 20N). After each filtration step, a sample was withdrawn for analysis of infectious virus particles and viral RNA. All filtrations were performed in dead-end mode with a pressure of 1 bar. Filter loads were 40 L/m^2^. Virus titer was determined by RT-PCR. Hold controls were titrated at the end of each process run.

### 2.7. Electron Microscopy

Virus-producing Vero cells were fixed with 2.5% glutaraldehyde in culture medium for 45 min. After washing with PBS, cells were scraped off the culture flask, transferred to a centrifuge tube and gently mixed with 2% warm, liquid agarose. The suspension chilled at 4 °C to harden. The agarose containing the suspended cells was cut into small pieces and then treated like small tissue blocks. These blocks were post-fixed with 2% osmium tetroxide in PBS, followed by 1% tannic acid and subsequently dehydrated in a graded series of ethanol and finally embedded in Agar 100 resin (Plano, Marburg, Germany) using standard procedures. Ultrathin sections were cut and stained with 2% uranyl acetate for 15 min followed by 2% lead citrate for 5 min. All specimens were examined under a JEM-1400 Flash electron microscope (JEOL) and micrographs were taken with a Xarosa digital camera (EMSIS). For measurement of viral size, pictures were taken under controlled and equal conditions of EM setting shortly after calibration of magnification. At least 75 particles were measured with Radius software (EMSIS) and the mean was calculated.

## 3. Results

### 3.1. Virus Inactivation by Heat Treatment

**Figure 1:**
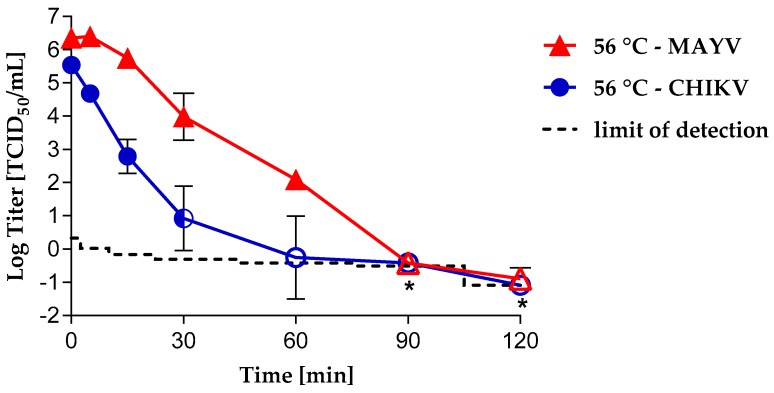
Heat inactivation kinetics of CHIKV and MAYV by heat treatment of cell culture medium. Heat-treated samples (mean values from triplicate runs) are indicated by circular (CHIKV) or triangular (MAYV) symbols. Error bars represent the standard deviation. *CHIKV data from one experiment; closed symbols = infectivity was detected; open symbols = no residual infectivity was detected; half-open symbol = in 2/3 experiments, residual infectivity was detected; the limit of detection depends on the sample volume tested.

We evaluated heat inactivation kinetics for CHIKV and MAYV under different conditions. In cell culture medium, the CHIKV heat inactivation kinetics showed a reduction of 2.74 log10 after only 15 min of heat treatment at 56 °C (Figure 1).

After 60 min, only a single well was found to be positive (i.e., 1/192 wells) in one out of three experiments using a large volume-plating assay. After 90 and 120 min of treatment, no virus was detected, indicating complete inactivation; a detection limit of −0.42 and −1.09 TCID_50_/mL was calculated, resulting in logarithmic reduction values (LRVs) of at least 5.73 and 6.4. In contrast, it took 30 min to achieve a 2.37 log10 reduction factor for MAYV. Complete inactivation was found only after 90 min of heat treatment, thus showing MAYV to be more resistant in medium than CHIKV (Figure 1). The detection limits were calculated for the samples taken after 90 and 120 min of heat treatment with −0.42 and ≤−1.09 TCID_50_/mL, resulting in logarithmic reduction factors of ≥6.77 and ≥7.25, respectively.

No decrease in infectivity of either CHIKV or MAYV was observed in the control samples held at room temperature until the end of experiment (Figure S1).

**Figure 2:**
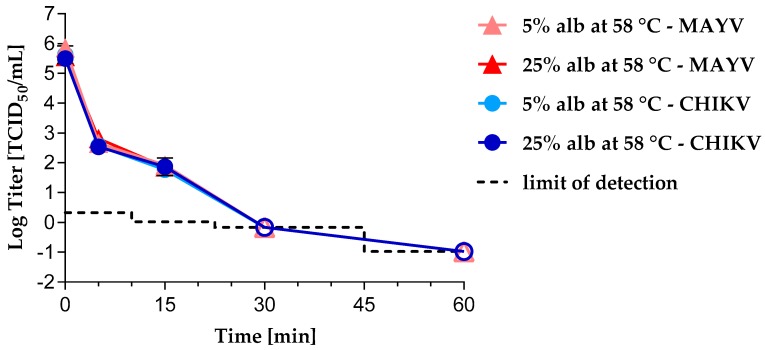
Heat inactivation kinetics of CHIKV and MAYV at 58 °C in 5% and 25% albumin solution. Heat-treated samples are indicated by circular (CHIKV) or triangular (MAYV) symbols. All experiments were performed in duplicate except the CHIKV treatment in 25% albumin solution (*n* = 3). Error bars represent the standard deviation. Alb = albumin; closed symbols = infectivity was detected; open symbols = no residual infectivity was detected; the limit of detection depends on the sample volume tested.

During manufacture of PDMPs such as human albumin, adventitious viruses are inactivated by pasteurization—10 h at 60 °C ± 1 °C. To investigate the virus inactivation profile of CHIKV and MAYV in PDMPs by pasteurization in a worst case scenario, we performed experiments at 58 °C in 5% albumin or 25% albumin. Irrespective of the albumin concentration, CHIKV and MAYV were inactivated after 30 min of heat treatment to a comparable extent (Figure 2).

For the CHIKV samples taken after 60 min of heat treatment, logarithmic reduction factors of 5.94 (5% albumin) and 6.48 (25% albumin) were observed. For the MAYV samples taken after 60 min of heat treatment, logarithmic reduction factors of 6.49 (5% albumin) and 6.54 (25% albumin) were observed. No decrease in infectivity of either CHIKV or MAYV was observed in the control samples held at room temperature until the end of experiment (Figure S2).

### 3.2. Virus Inactivation byS/D Treatment

**Figure 3:**
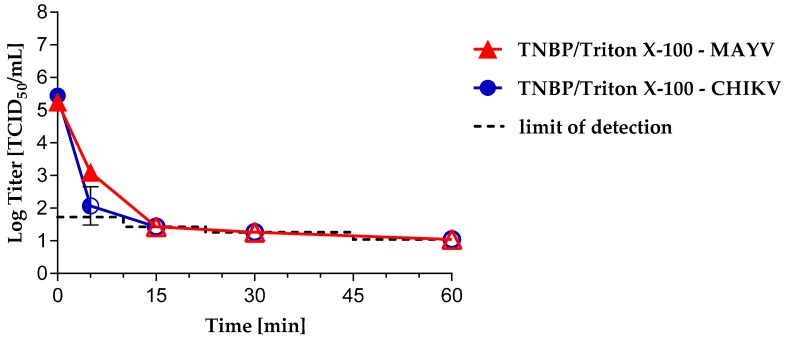
Inactivation kinetics of CHIKV and MAYV under S/D treatment using 0.3% TNBP/1% Triton X-100. CHIKV or MAYV were spiked into 5% albumin solution containing S/D reagents. Experiments were performed in triplicate. Error bars represent the standard deviation. S/D treated samples are indicated by circular (CHIKV) or triangular (MAYV) symbols. Closed symbols = infectivity was detected; open symbols = no residual infectivity was detected; half-open symbol = in 1/3 experiments residual infectivity was detected; the limit of detection depends on the sample volume assayed.

TNBP/Triton X-100, TNBP/Tween 80 or TNBP/sodium deoxycholate are commonly used S/D mixtures in the manufacturing process of PDMPs. We investigated the inactivation profiles of CHIKV or MAYV by using final concentrations of 0.3% TNBP/1% Triton X-100, 0.3% TNBP/1% Tween 80 or 0.3% TNBP/0.2% sodium deoxycholate. Using TNBP/Triton X-100, CHIKV and MAYV were completely inactivated after 15 min of S/D treatment (Figure 3). Maximal logarithmic reduction values of ≥4.38 (CHIKV) and ≥4.16 (MAYV) were observed.

**Figure 4:**
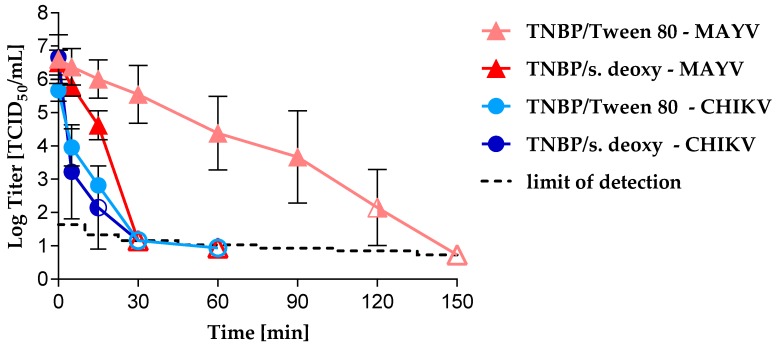
Inactivation kinetics of CHIKV and MAYV under S/D treatment using 0.3% TNBP/1% Tween 80 or 0.3% TNBP/0.2% sodium deoxycholate. CHIKV or MAYV were spiked into 5% albumin solution containing S/D reagents. All experiments were performed in triplicate except the MAYV S/D treatment using TNBP/Tween 80 (*n* = 8). Error bars represent the standard deviation. S/D-treated samples are indicated by circular (CHIKV) or triangular (MAYV) symbols. Closed symbols = infectivity was detected; open symbols = no residual infectivity was detected; half-open symbol = in 4/8 (MAYV) or 1/3 (CHIKV) experiments, residual infectivity was detected; the limit of detection depends on the sample volume assayed.

Using either TNBP/Tween 80 or TNBP/deoxycholate, CHIKV was completely inactivated after 30 min of S/D treatment (Figure 4).

After 15 min of TNBP/sodium deoxycholate treatment, no residual CHIKV infectivity was detected in two out of three experiments. After 15 min incubation with TNBP/Tween 80, CHIKV infectivity was detected in all three experiments (LRV = 2.86). Maximal logarithmic reduction values of ≥4.73 (TNBP/Tween 80) and ≥5.72 (TNBP/sodium deoxycholate) were observed. In contrast, complete inactivation of MAYV was only observed after 2.5 h of TNBP/Tween 80 treatment and after 30 min of TNBP/sodium deoxycholate treatment (Figure 4). A maximum LRV of ≥5.88 was calculated for treatment with TNBP/Tween 80 and of ≥5.72 for treatment with TNBP/sodium deoxycholate. Bench control samples showed no decrease of infectivity for either CHIKV or MAYV over the period of all S/D treatment experiments (Figure S3 and S4).

### 3.3. Virus Removal by Nanofiltration

We studied virus removal by nanofiltration using virus filters with different pore diameters: 0.5% albumin solution was spiked with CHIKV or MAYV, filtered through a 0.1 µm filter in order to remove virus aggregates. Subsequently, supernatants were filtered through filters with 20 nm pore diameter (Run 1) and 35 nm pore diameter (Run 2). No CHIKV or MAYV infectivity could be detected in either of the filtrates (Table 1).

However, after 75 nm filtration (Run 3), a small quantity of infectivity (1.01 log10 TCID_50_/mL MAYV and 1.09 log10 TCID_50_/mL CHIKV) was detected by TCID_50_ assay. In addition, a series of filters with decreasing pore diameters, namely, 75 nm, 40 nm, 35 nm, and 20 nm (Run 4) were used for filtration. Samples were withdrawn after each filtration step and titrated. Complete removal of CHIKV as well as MAYV infectious particles was demonstrated already after the first 75 nm filtration step. In addition to the infectivity assay, we performed quantitative RT-PCR analysis of the CHIKV samples using the WHO 1st CHIKV international standard to provide information about the RNA content of the filtrates and thus the relation between infectious particles and measured RNA units. RNA contents turned out to be 1000-fold higher than the detected amount of infectious particles in the spiked albumin solution or pre-filtrates. However, decreasing amounts of viral RNA were detected concomitant with the use of smaller pore diameter filters. Whereas in the 75 nm filtrates, loads of 3.49 and 3.73 log10 IU/mL CHIKV RNA were detected, analysis of the 40 nm and 35 nm filtrates derived from run 2 and run 4 showed a viral RNA content of 2.27–2.53 log10 IU/mL. Filtrates from the 20 nm filter contained no detectable viral RNA. LRFs from all inactivation/removal experiments are listed in Table S1.

### 3.4. Electron Microscopy

**Figure 5:**
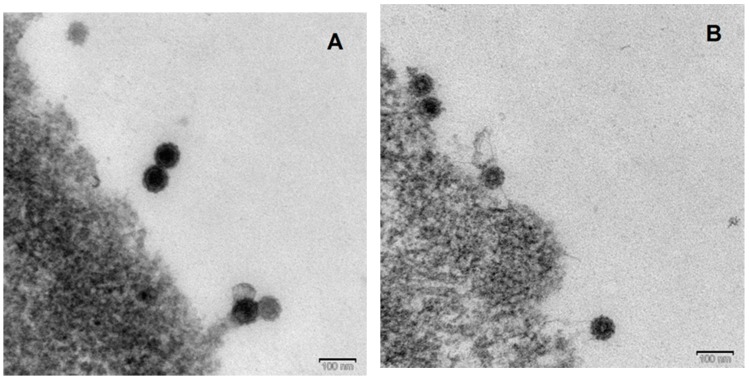
Ultrathin sections of (**A**) CHIKV or (**B**) MAYV infected Vero cells. Round enveloped particles with well visible spikes with an average diameter of 66–67 nm were detected.

We compared the size of the CHIKV and MAYV particles by examination of stained ultrathin sections of infected Vero cells using a JEM-1400 Flash electron microscope. Electron micrographs were taken (Figure 5) and the size of particles was measured using the Radius software (EMSIS).

The mean of at least 75 particles was calculated, resulting in a virion size of 67.17 ± 3.62 nm for CHIKV and 66.4 ± 3.46 nm for MAYV. The difference was statistically not significant.

## 4. Discussion

Safety of plasma-derived medicinal products with respect to emerging viruses can be achieved by a combination of various methods for inactivation or removal of viruses, such as precipitation with ethanol, heat treatment, S/D treatment or nanofiltration [36]. Little is known about the reduction capacities of inactivation/removal procedures for alphaviruses. Previous studies demonstrated temperature tolerance of CHIKV and other alphaviruses such as Western Equine Encephalitis virus and Ross River virus at 56 °C [24,37,38,39]. In this study we investigated the efficiency of heat treatment as well as S/D treatment to inactivate MAYV or CHIKV and determined the inactivation kinetics. In addition, we examined the effectivity of virus removal filtration using filters with average pore diameter of 75 nm, 40 nm, 35 nm, and 20 nm.

In agreement with previous studies, in which CHIKV-spiked cell culture medium supplemented with serum was heat treated [26,37], incomplete inactivation was observed after 30 min at 56 °C. Surprisingly, the results showed MAYV to be somewhat more temperature resistant than CHIKV despite being of same size and phylogenic closely related. This suggests structural differences between the viruses. Nevertheless, after 90 min at 56 °C, both viruses were completely inactivated. The fact that the heat inactivation kinetics can differ between members of the same virus family and even within the same genus has already been shown in studies with Flaviviruses, such as ZIKV, WNV or Bovine viral diarrhea virus, which are structurally and functionally related to alphaviruses [40,41]. Our results underline the importance of appropriate controls and extended heat inactivation time of samples in serological tests, which eliminate the risk of false-negative results caused by residual infectivity in serum/plasma samples. Moreover, special care should be taken during handling of supposedly virus-inactivated patient samples when performing serological diagnostics.

Pasteurization—60 °C for 10 h—is an effective inactivation step for enveloped and some non-enveloped viruses during manufacturing of PDMPs. To address the question of chikungunya or Mayaro viral safety of PDMPs by pasteurization in a worst case scenario, we performed heat inactivation of CHIKV or MAYV in 5% or 25% HSA solution as a stabilizing agent at 58 °C. Irrespective of the albumin content, both, CHIKV and MAYV were completely inactivated after 30 min of heat treatment. Our results confirm that pasteurization is a reliable and robust inactivation method even for rather temperature-resistant enveloped viruses such as MAYV.

Espíndola et al. investigated the efficiency of heat treatment to inactivate SINV, another member of the genus *alphavirus*, in a human factor VIII product. Similar to our findings with CHIKV, heat treatment was shown to effectively inactivate SINV in a semi-processed VIII concentrate. Remarkably, the inactivation of SINV was far less efficient in lyophilized and reconstituted VIII concentrates when compared to PBS controls. Thus, the results emphazise the relevance of testing stabilizing agents in inactivation studies [25].

We investigated the inactivation kinetics of CHIKV or MAYV by S/D treatment under conditions commonly used in the manufacture of PDMPs: Final concentrations of 0.3% TNBP/1% Triton X-100 or 0.3% TNBP/1% Tween 80 or 0.3% TNBP/0.2% sodium deoxycholate. As expected from many other studies [42], inactivation kinetics were most rapid using the combination of Triton X-100/TNBP. Both CHIKV and MAVV were inactivated below the detection limit in 15 min. However, because of environmental protection concerns the use of Triton X-100 during commercial manufacturing is being restricted in the European Union [43]. Therefore, there is increasing interest in alternative detergents. In our study, CHIKV was also completely inactivated using TNBP/sodium deoxycholate or TNBP/Tween 80 after 30 min incubation. Even though MAYV and CHIKV membrane composition would be expected to be very similar, MAYV was shown to be more resistant to TNBP/sodium deoxycholate after 15 min incubation, but was nevertheless completely inactivated after 30 min. In contrast, the rate of MAYV inactivation was unexpectedly slow using TNBP/Tween 80. Only after 2.5 h of TNBP/Tween 80 treatment was MAYV completely inactivated with no evidence of residual infectivity. This phenomenon agrees with a study showing inactivation of vaccinia virus spiked process intermediates of anti-hemophilic factor (AHF, Koate^®^-DVI) by TNBP/cholate being more efficient than using TNBP/Tween 80 [40]. Another study investigated the inactivation kinetics of enveloped viruses of different virus families, including SINV and Vaccinia virus, in different process intermediates such as factor IX, using various S/D mixtures. Using 0.3% TNBP/1% Tween 80, vaccinia viral infectivity was inefficiently reduced after 6 h incubation, whereas SINV was efficiently inactivated after just 30 min incubation [44]. In contrast, a recent study demonstrates the variability of inactivation efficiencies of S/D treatment of, amongst others, alphaviruses and vesiculoviruses: SINV or vesicular stomatitis virus (VSV) were inactivated in AHF concentrate with 0.3% TNBP/0.2% sodium cholate or 0.3% TNBP/1% Tween 80. Surprisingly, the inactivation by TNBP/Tween 80 was more efficient as compared to TNBP/sodium deoxycholate for both viruses. SINV was inactivated to an undetectable level after 15 min with TNBP/Tween 80 and after 2 h with TNBP/sodium deoxycholate [23]. A meta-analysis investigating the robustness of S/D treatment of plasma derivatives revealed some low LRVs for low concentrations of TNBP in combination with Tween 80 in some studies. However, it was concluded that these outliers are due to a larger number of studies and associated greater variance of LRVs compared to the lower number of studies investigating other combinations of S/D. Based on this meta-analysis, it was stated that pH, product class, protein concentration, and low incubation temperature do not strongly affect the virus inactivation capacity, but short incubation times undoubtedly result in a larger range of LRVs [42]. Although in our study, inactivation of MAYV was unexpectedly delayed up to 2.5 h using the Tween/TNBP combination, the usual duration of treatment for PDMPs is 6 h and this process can be considered to inactivate a broad variety of enveloped viruses. Nevertheless, the difference poses the question of the use of model viruses for S/D treatment. BVDV and Pseudorabies virus (PRV) are commonly used as model viruses. In an earlier study, we extensively investigated inactivation kinetics of BVDV, PRV, SFV and VSV in 1% Tween 80/0.3% TNPB [24]. All four models were inactivated below the detection limit after 15–60 min which fits to the inactivation of CHIKV observed in this study. In summary, the greater resistance of MAYV raises questions about differences in MAYV and CHIKV particle assembly and composition and emphasizes the importance of performing virus reduction studies using a wider panel of viruses and a thorough investigation of inactivation kinetics, including under robustness conditions.

Virus removal by nanofiltration (e.g., 35 nm or 20 nm) is an essential step in the downstream process of biopharmaceuticals to ensure viral and prion safety. Depending on the size of the product to be purified, filters with average pore diameters ranging from 15–35 nm are used while 75 nm filters may be used as pre-filters [45,46]. We investigated the virus removal capacity of 75 nm, 35 nm, and 20 nm filters. Due to the same size of CHIKV and MAYV particles of 66–67 nm, which we determined by electron microscopy, we anticipated some virus removal capacity of the 75 nm filters and strong removal capacity of the filters with smaller pore diameters. As expected, MAYV and CHIKV were removed completely by the 40 nm, 35 nm, and 20 nm filters. The 75 nm filters substantially reduced viral particles almost to the limit of detection. These findings were reflected by the detection of viral RNA in the filtrates. The amount of viral RNA in the filtrates was reduced with decreasing pore diameter. Nevertheless, viral sequences were detected in the 75 nm, 40 nm, and 35 nm filtrates. However, during virus propagation in cell culture, viral genomes are commonly overproduced with only a small proportion being packaged into infectious particles. For this reason, we performed a nuclease digestion prior to the RNA extraction which should remove unpackaged viral RNA from the supernatants. The presence of high amounts of viral genome RNA concomitant with the absence of detectable infectious particles in the filtrates is most likely caused by an incomplete digestion of viral RNA due to complexed viral proteins and by the exceeding amount of viral RNA present in the cell culture supernatant. For Flaviviruses, Arenaviruses, Bunyaviruses, and Filoviruses, ratios of viral RNA to infectious particles ranging from 1:1 to 8.6 × 10^7^:1 in cell culture were determined [47]. As we previously showed for ZIKV [34], only the 20 nm filter seems to be capable to remove residual complexed virus RNA.

## 5. Conclusions

In summary, we demonstrate that even closely related alphaviruses within the Semliki Forest complex behave differently under heat or detergent treatment. However, we confirm that the downstream processes commonly used in the blood product industry are capable of effectively inactivating/removing the newly emerging MAYV and CHIKV.

## Figures and Tables

**Table 1:** Reduction of viral particles and viral RNA content by nanofiltration.

Run	Sample Description	MAYV Infectivity [log TCID_50_/mL ± 95% CI]	CHIKV Infectivity [log TCID_50_/mL ± 95% CI]	CHIKV RNA [log IU/mL]
1) 20 nm filter	Spiked load	5.99 ± 0.30	5.45 ± 0.24	n.d.
	0.1 µm pre-filtrate	5.79 ± 0.30	5.28 ± 0.24	8.62
	20 nm filtrate	≤−0.41	≤−0.41	-
2) 35 nm filter	Spiked load	5.79 ± 0.30	5.55 ± 0.24	n.d.
	0.1 µm pre-filtrate	5.61 ± 0.30	5.34 ± 0.24	8.62
	35 nm filtrate	≤0.41	≤−0.41	2.29
3) 75 nm filter	Spiked load	6.51 ± 0.33	5.65 ± 0.35	8.87
	0.1 µm pre-filtrate	6.29 ± 0.33	5.43 ± 0.35	n.d.
	75 nm filtrate	1.01 ± 0.49	1.09 ± 0.47	3.49
4) 75, 40, 35, 20 nm filter	Spiked load	6.31 ± 0.30	5.37 ± 0.24	8.72
	75 nm filtrate	≤1.03	≤1.03	3.73
	40 nm filtrate	≤−0.4	≤−0.4	2.53
	35 nm filtrate	≤−0.72	≤−0.72	2.27
	20 nm filtrate	≤−0.73	≤−0.73	-

n.d. = not determined; - = not detected; IU = international units; CI = confidence interval.

## Supplementary Materials

The following are available online at https://www.mdpi.com/1999-4915/11/3/234/s1, Figure S1: Hold controls of heat inactivation kinetics of CHIKV and MAYV by heat treatment of cell culture medium, Figure S2: Hold controls of heat inactivation kinetics of CHIKV and MAYV at 58 °C in 5% and 25% albumin solution, Figure S3: Bench controls of inactivation kinetics of CHIKV and MAYV under S/D treatment using 0.3% TNBP/1% Triton X-100, Figure S4: Bench controls of inactivation kinetics of CHIKV and MAYV under S/D treatment using 0.3% TNBP/1% Tween 80 or 0.3% TNBP/0.2% sodium deoxycholate, Table S1: Log10 reduction factors (LRFs) from the inactivation and removal experiments.
