# Supplementary material for: Inactivation and Removal of Chikungunya Virus and Mayaro Virus from Plasma-derived Medicinal Products
Source: Viruses. 2019 Mar 7;11(3):234. doi: 10.3390/v11030234 (PMC6466239; doi:10.3390/v11030234)
Supplement: Supplementary file 1 [file viruses-11-00234-s001.zip › Supplemental Table 2.docx]

**Table 2.** Log_10_ reduction factors (LRFs) from the inactivation and removal experiments

| **Method of**  **Inactivation/**  **Removal** | | | **LRF CHIKV**  **30 min** | **LRF CHIKV**  **60 min/**  **filtrate** | **LRF MAYV**  **30 min** | **LRF MAYV**  **60 min/**  **filtrate** |
| --- | --- | --- | --- | --- | --- | --- |
| **Heat** | 56°C | | 4.61 | ≥5.79 | 2.37 | 4.26 |
|  | 58°C/ 5% alb | | ≥5.8 | ≥5.94 | ≥6.02 | ≥6.49 |
|  | 58°C/ 25% alb | | ≥5.67 | ≥6.48 | ≥5.73 | ≥6.54 |
| **S/D** | TNBP/Triton X-100 | | ≥4.12 | ≥4.34 | ≥3.94 | ≥4.16 |
|  | TNBP/Tween 80 | | ≥4.51 | ≥4.73 | 1.06 | 2.22 |
|  | TNBP/s. deoxy | | ≥5.5 | ≥5.72 | ≥5.53 | ≥5.72 |
| **Nanofiltration** | **Run 1** | 20 nm filter | n.a. | ≥5.69 | n.a. | ≥6.2 |
|  | **Run 2** | 35 nm filter | n.a. | ≥5.75 | n.a. | ≥6.02 |
|  | **Run 3** | 75 nm filter | n.a. | 4.34 | n.a. | 5.28 |
|  | **Run 4** | 75 nm filter | n.a. | ≥4.34 | n.a. | ≥5.28 |
|  |  | 40 nm filter | n.a. | ≥5.77 | n.a. | ≥6.71 |
|  |  | 35 nm filter | n.a. | ≥6.09 | n.a. | ≥7.03 |
|  |  | 20 nm filter | n.a. | ≥6.1 | n.a. | ≥7.04 |

alb = albumin; s. deoxy = sodium deoxycholate; n.a. = not applicable
