# Supplementary figures and images for: Inactivation and Removal of Chikungunya Virus and Mayaro Virus from Plasma-derived Medicinal Products
Source: Viruses. 2019 Mar 7;11(3):234. doi: 10.3390/v11030234 (PMC6466239; doi:10.3390/v11030234)

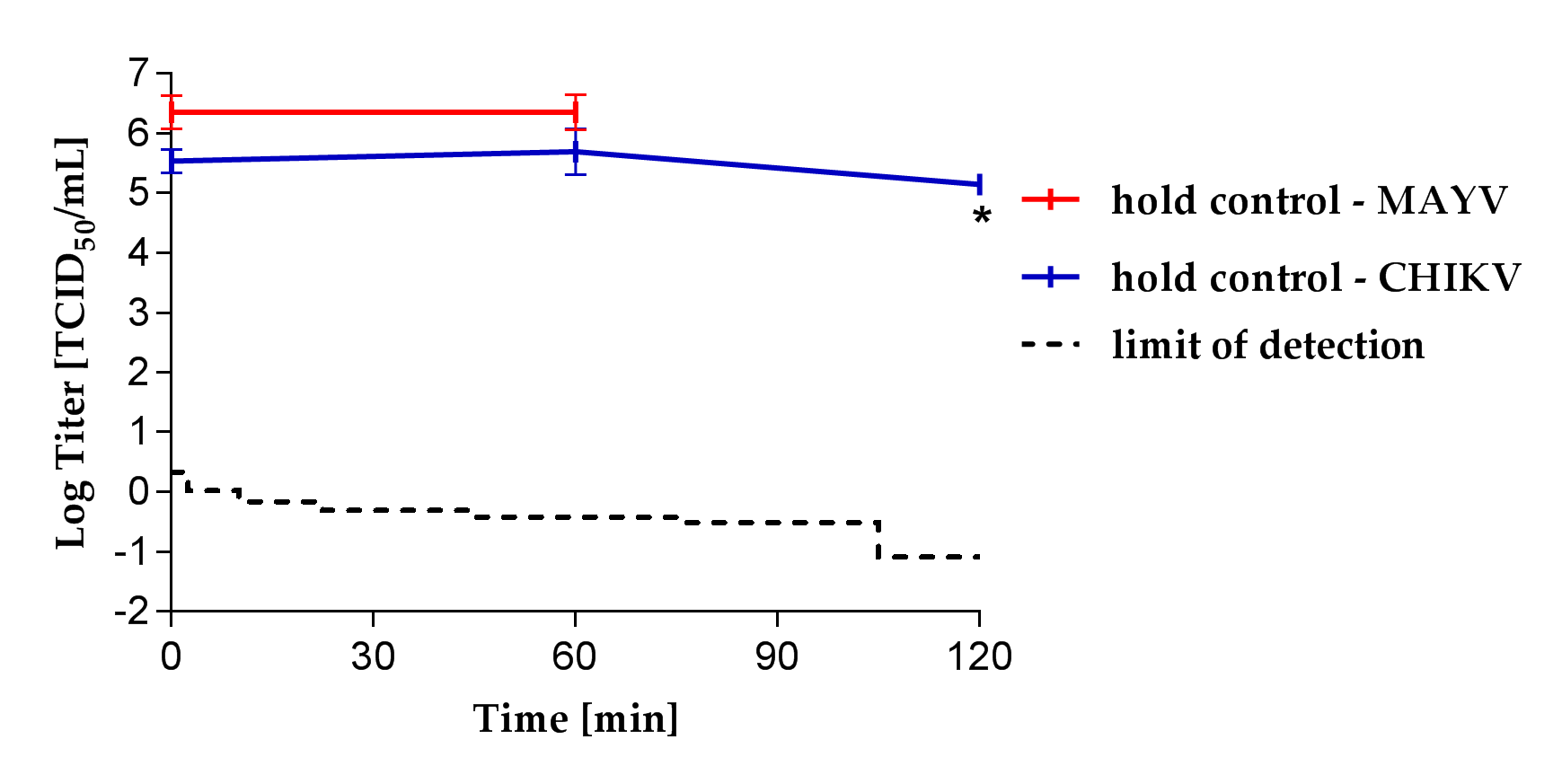

Supplement: Supplementary file 1 [file viruses-11-00234-s001.zip › Supplemental Figure 1a.tif]

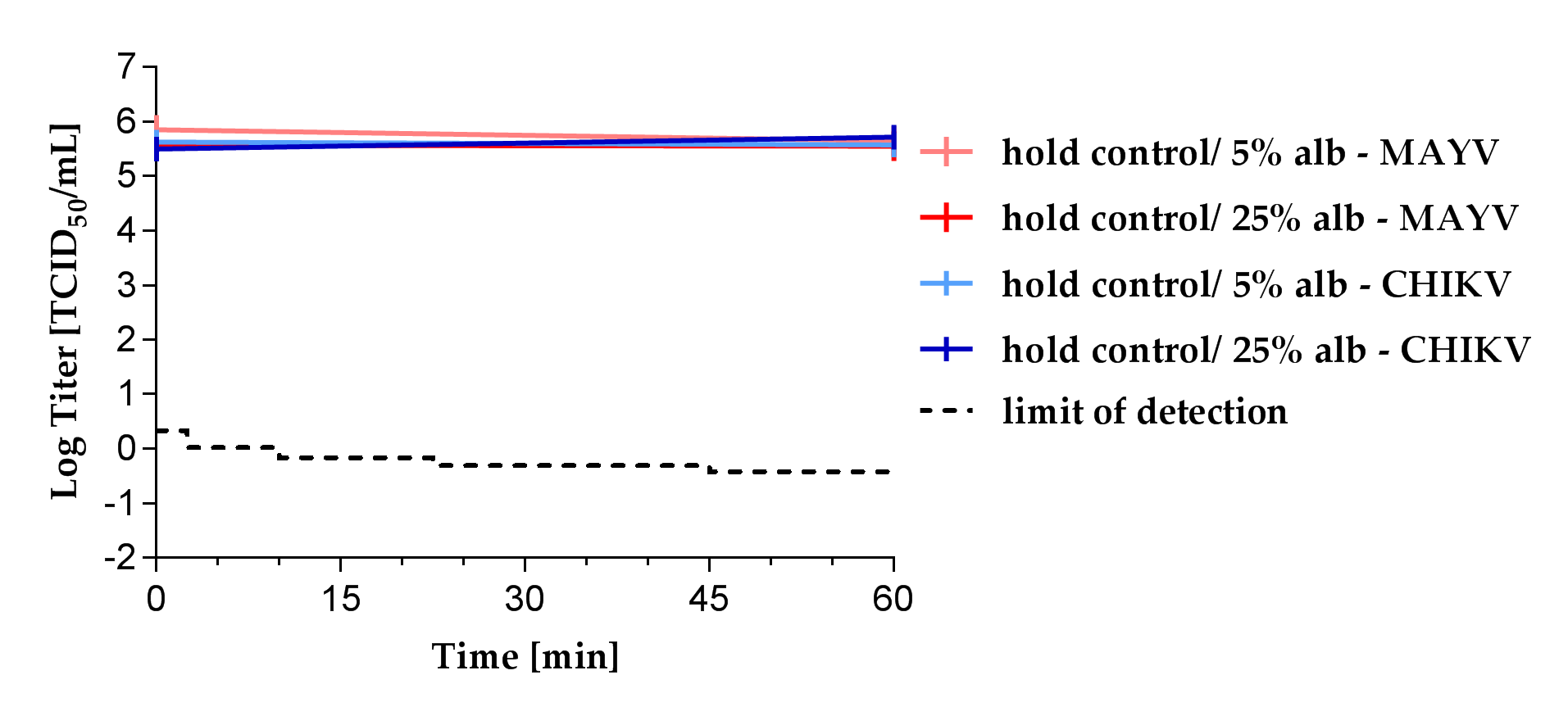

Supplement: Supplementary file 1 [file viruses-11-00234-s001.zip › Supplemental Figure 2a.tif]

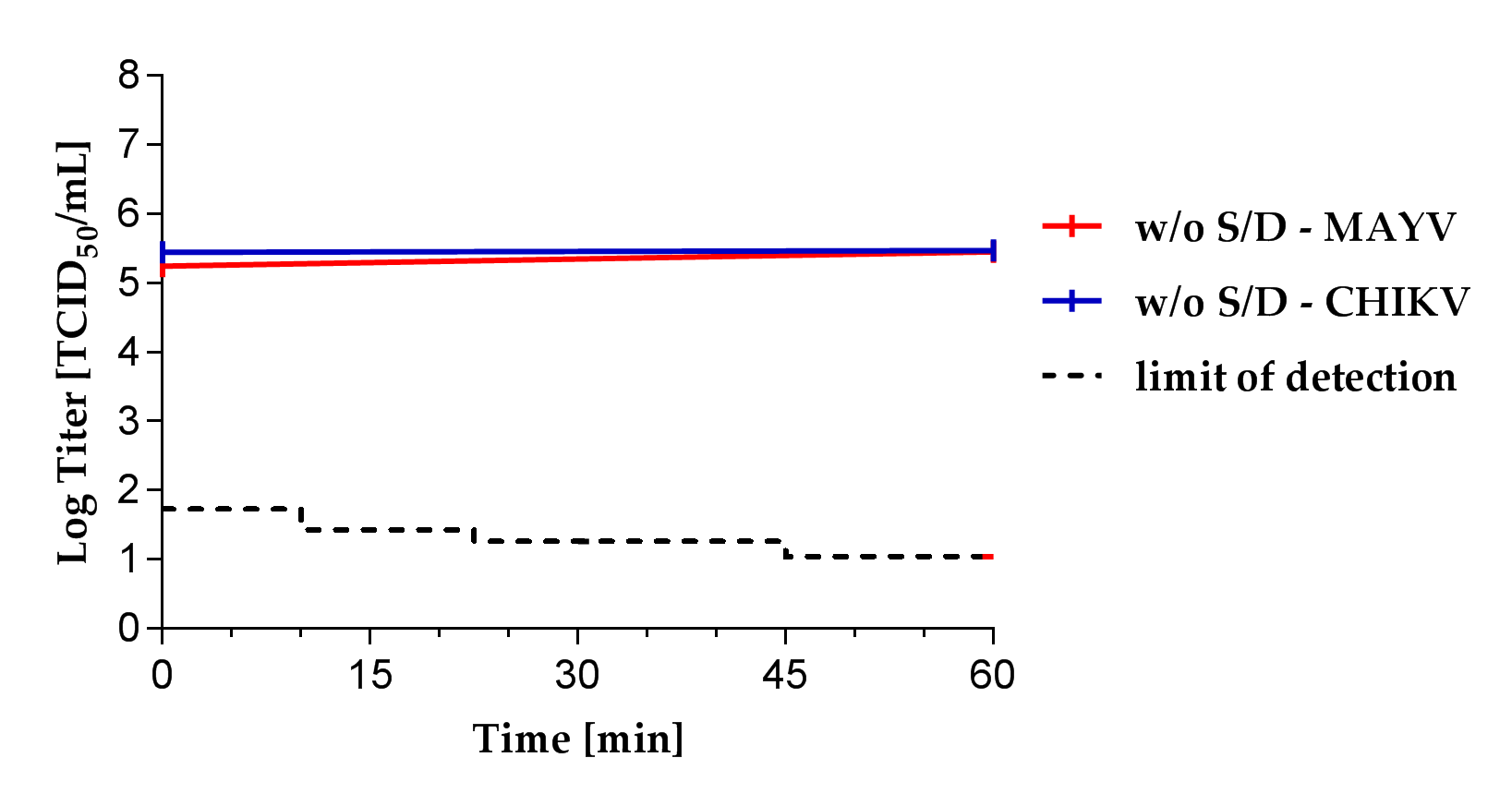

Supplement: Supplementary file 1 [file viruses-11-00234-s001.zip › Supplemental Figure 3a.tif]

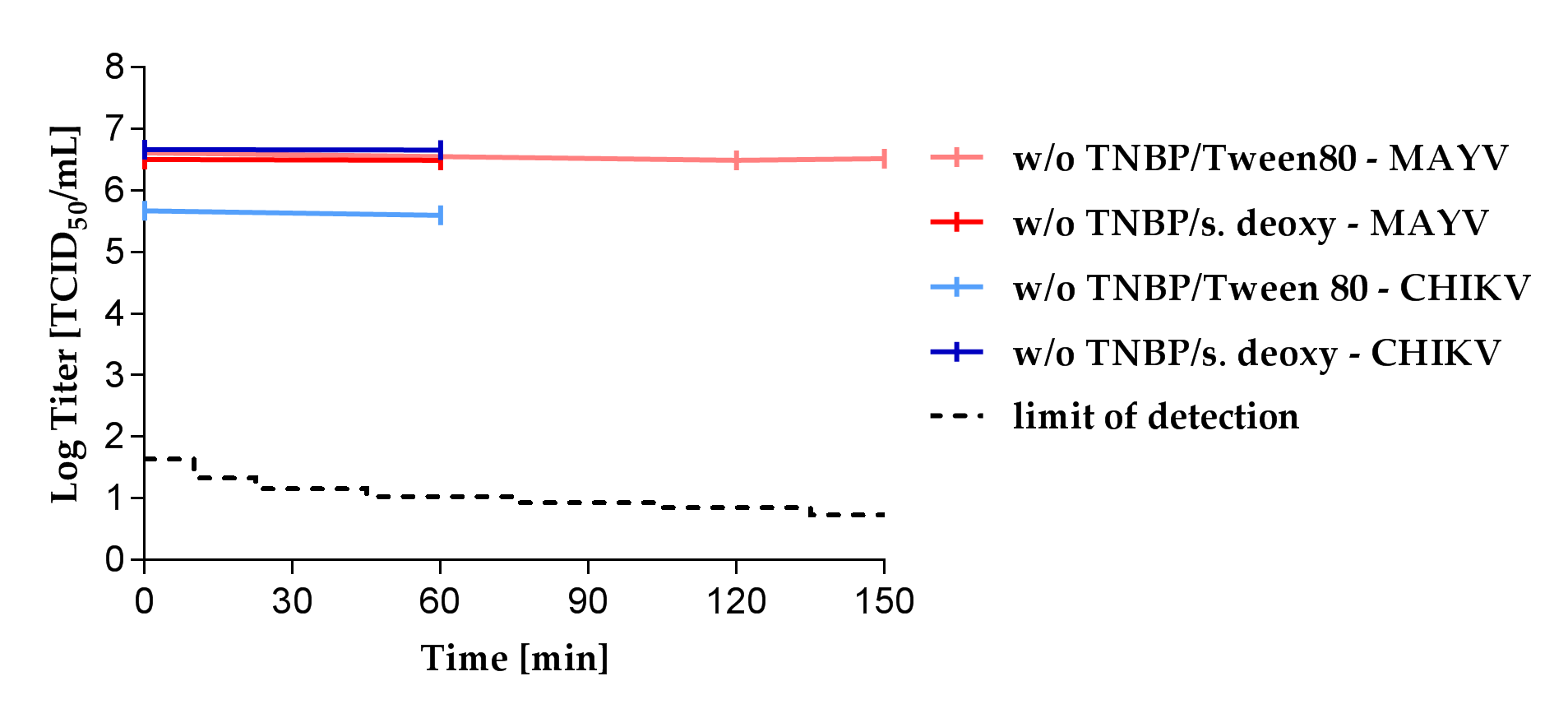

Supplement: Supplementary file 1 [file viruses-11-00234-s001.zip › Supplemental Figure 4a.tif]
